# Potential Clinical Application of Genomics in Multiple Myeloma

**DOI:** 10.3390/ijms19061721

**Published:** 2018-06-10

**Authors:** Cinnie Yentia Soekojo, Sanjay de Mel, Melissa Ooi, Benedict Yan, Wee Joo Chng

**Affiliations:** 1 Department of Hematology-Oncology, National University Cancer Institute, Singapore, National University Health System, 1E Kent Ridge Road, Singapore 119228, Singapore; cinnie_yentia_soekojo@nuhs.edu.sg (C.Y.S.); sanjay_widanalage@nuhs.edu.sg (S.d.M.); melissa_ooi@nuhs.edu.sg (M.O.); 2 Department of Laboratory Medicine, National University Hospital, National University Health System, 5 Lower Kent Ridge Road, Singapore 119074, Singapore; benedict_yan@nuhs.edu.sg; 3 Cancer Science Institute of Singapore, National University of Singapore,14 Medical Drive, Singapore 117599, Singapore

**Keywords:** multiple myeloma, genomics, gene expression profiling

## Abstract

Multiple myeloma is a heterogeneous disease with different characteristics, and genetic aberrations play important roles in this heterogeneity. Studies have shown that these genetic aberrations are crucial in prognostication and response assessment; recent efforts have focused on their possible therapeutic implications. Despite many emerging studies being published, the best way to incorporate these results into clinical practice remains unclear. In this review paper we describe the different genomic techniques available, including the latest advancements, and discuss the potential clinical application of genomics in multiple myeloma.

## 1. Introduction

Multiple myeloma (MM) is a malignancy arising from post-germinal B-cells characterized by the proliferation of terminally differentiated clonal plasma cells in the bone marrow. These cells secrete a monoclonal protein that can be measured in the serum and urine. Patients present with symptoms or end organ damage such as anemia, hypercalcemia, bone pain, or renal impairment. It is the second most common hematological malignancy in adults in the world.

As with other malignancies, much has been learnt about myeloma biology from genetic studies. More importantly, genetic techniques have evolved and the utility of genetic information has important clinical impact. In this review, we examine the different techniques and the clinical utility of genomic information in myeloma. We searched PubMed for studies that evaluate genomics in MM, using search terms including “genomics”, “myeloma”, “next generation sequencing”, “mutations”, “gene expression”, “microarray”, “prognosis”, “minimal residual disease (MRD)”, and manually curated the papers that were relevant.

## 2. Genetic and Genomic Techniques in Multiple Myeloma

### 2.1. Metaphase Karyotyping

Karyotyping was the earliest technique used to study the MM genome and remains an important part of clinical practice today [1]. This technique involves the culture of bone marrow cells followed by the harvest of metaphase chromosomes and induction of mitotic arrest with colcemid or colchicine treatment [2]. The most common technique used for chromosome staining is G-banding, which involves the treatment of the chromosomes with trypsin followed by Giemsa staining [3]. The stained chromosomes are then analyzed using light microscopy by a cytogeneticist [2].

Karyotyping is helpful in identifying numerical as well as structural chromosomal abnormalities [2]. The initiating genetic events in MM are immunoglobulin heavy chain (IgH) translocations or hyperdiploidy(characterized by odd chromosome trisomies), both of which can be detected using karyotyping [4,5,6]. Structural abnormalities of significance that can be detected by karyotyping include the 17p and 13q deletions along with amplification of 1q21 [6]. It is noteworthy that numeric abnormalities involving these chromosomes such as monosomy 17 or 13 can also occur [6].

Although karyotyping is widely available and affordable, an important drawback of this technique is that the presence of plasma cells in metaphase is mandatory to be able to detect an abnormality [6]. As MM is usually associated with a low proliferation rate, it is not surprising that only 25–30% of MM patients have genetic abnormalities detected by karyotyping [7]. It has therefore been proposed that the prognostic significance of karyotyping can be at least partly attributed to the high proliferative index in patients who have detectable abnormalities [6]. Modifications of conventional karyotyping such as spectral karyotyping (SKY) have shown some utility in evaluating MM with complex karyotypes [8]. They are, however, not in mainstream clinical use. More sensitive techniques with higher resolution have therefore been developed to better understand the genetic abnormalities in MM.

### 2.2. Fluorescent In Situ Hybridization (FISH)

FISH involves the use of DNA probes with a fluorescent label that hybridizes with the probes and allows the identification of specific DNA sequences in the genome [2]. There are two broad categories of FISH probes used in routine clinical practice for MM, locus-specific identifier (LSI) probes and centromeric enumeration probes (CEP) [2]. LSI probes consist of two subtypes: break apart and fusion probes. Break apart probes bind to loci on either side of a specific locus and are helpful in detecting gene rearrangements where the partner gene is unknown or in identifying gene deletions [2]. *MYC* rearrangement is an example where break apart probes are useful in MM [2,6]. LSI are also important for the detection of 17p deletion, which has significant prognostic significance in MM [6]. Dual color fusion probes bind to specific loci on known genes involved in a translocation; for example, the IgH translocations in MM can be detected by this method [2,6]. CEP bind to alpha satellite repeat sequences on the centromeres of each chromosome and are helpful for chromosome enumeration [2]. They are useful for the identification of numerical chromosome abnormalities such as hyperdiploidy and monosomy 17 in MM [6]. The value of FISH in chromosome enumeration was highlighted by a study demonstrating that trisomies of chromosomes 9, 11, and 15 as assessed by FISH had a strong predictive power for differentiating hyperdiploid versus nonhyperdiploid monoclonal gammopathy of undetermined significance (MGUS) [9].

The International Myeloma Working Group (IMWG) and European Myeloma Network (EMN) recommend that FISH be performed on plasma cells sorted from other bone marrow (BM) cells by cytoplasmic immunoglobulin expression (cIg FISH) [10,11]. cIg FISH results in higher specificity and sensitivity compared to conventional FISH, especially in cases where the plasma cells comprise a low percentage of nucleated cells in the BM [12]. Simultaneous staining of the plasma cells with antibodies against cytoplasmic immunoglobulin followed by FISH is the most commonly used technique, while flow cytometry and immunomagnetic beads have also been used for this purpose [12,13].

In view of its high sensitivity and applicability in interphase plasma cells, FISH is the standard of care for the assessment of MM-related cytogenetic abnormalities in clinical practice [6,14]. Although highly effective as a clinical tool, a drawback of FISH is that it can only detect known recurrent abnormalities and cannot identify novel or unexpected genetic changes [15]. Variants of conventional FISH such as multicolor FISH have shown utility in detecting cryptic chromosomal abnormalities in MM with complex karyotypes [16]. These techniques are, however, yet to be widely applied in clinical practice.

### 2.3. Gene Expression Profiling and RNA Sequencing

Gene expression profiling (GEP) is a powerful tool for assessing the transcriptome of cancer cells [17]. This technique requires a human DNA microarray which is created using single-stranded nucleic acid probes covalently bound to a glass slide to create a gene chip. The mRNA from the cancer cell of interest is reverse transcribed into copy DNA (cDNA), which is then fluorescently labeled and hybridized with the single-stranded DNA on the chip [18]. The chip is then scanned and the differential gene expression in the cancer cell compared to the control cell can be evaluated by signal intensity [19].

RNA sequencing(RNASeq) requires RNA from the cancer cell of interest to be fragmented and labeled with adapter sequences prior to reverse transcription into cDNA, which is then sequenced in parallel multiple times [19]. RNASeq has multiple advantages over GEP; for example, splicing variants, noncoding RNA, and fusion proteins can be detected more readily by the former [20,21,22].

RNASeq is a promising avenue to augment our understanding of the pathogenesis and molecular biology of MM. In a study of 255 newly diagnosed MM patients, RNASeq identified novel fusion genes which were associated with clinical characteristics and outcome [23]. RNASeq signatures have also been proposed as a means to determine drug sensitivity, although further work is required before these techniques can be used clinically [24].

### 2.4. Array Comparative Genomic Hybridization

Array Comparative Genomic Hybridization (ACGH) is a useful technique for assessing genome-wide copy number changes in cancer. The initial step in ACGH is the creation of a DNA microarray which can be either representative of the whole genome or selected genes [25]. The specimen of interest and reference sample are then labeled with different fluorophores and equal amounts of test and reference sample are hybridized to the probes [26]. The fluorescence emission is proportional to the amount of DNA in the sample. The difference in fluorescence allows a comparison between the test and reference samples, allowing the identification of genomewide copy number changes [27].

In a study of transplant eligible MM patients, ACGH identified the loss of chromosome 1p31 as a significant predictor of shorter post-relapse survival [15]. Chromosome 1p deletion was also detected in 39% of patients in an ACGH study of 91 MM patients in whom the gain of chromosome 1q was reported as the most common copy number gain [28]. Indeed, chromosomal loss detected by ACGH has been reported as an independent prognostic factor in a cohort of Spanish MM patients [29]. Studies comparing karyotyping, ACGH and FISH on sorted plasma cells showed that ACGH had the greatest sensitivity for detecting genomic imbalances [30,31]. FISH was, however, superior for detecting IgH translocations [30]. These data suggest that ACGH and FISH (both performed on enriched plasma cells) could potentially be used together as tools for risk stratification in clinical practice [32]. ACGH has also been used in conjunction with next-generation sequencing to show that MGUS plasma cells have a lower number of somatic mutations and copy number alterations compared to MM plasma cells [33]. Furthermore, ACGH has been helpful in identifying novel candidate tumor suppressor genes in MM [34]. Taken together, these data indicate that ACGH is a promising tool for both clinical risk stratification and research in MM.

### 2.5. Next-Generation Sequencing

Next-generation sequencing (NGS) has resulted in rapid advances in our understanding of cancer genomics over the last decade [35]. The most commonly used NGS technique is “sequencing by synthesis”, which involves the DNA strand to be sequenced being used as a template to create a complimentary strand [36]. Fluorescently labeled nucleotides and optical imaging are used to visualize the growing complementary strand and hence establish the sequence of the DNA of interest, and this process is performed multiple times in parallel (massively parallel sequencing) [36]. Alternative methods include pyrosequencing, where a chemiluminescent signal is used to indicate base incorporation, and the ion torrent platform, where a change in pH resulting from nucleotide incorporation is detected on a semiconductor chip [36].

The advantages of NGS over Sanger sequencing include reduced turnaround time as a result of massively parallel sequencing and the smaller required volume of the test specimen [35]. Importantly, NGS is significantly more sensitive than Sanger sequencing [35]. NGS can be used to sequence the whole genome (whole genome sequencing or WGS), the coding regions of the genome (whole exome sequencing or WES), or a specific region of interest (targeted sequencing) [35]. RNASeq discussed above also employs the principles of NGS applied to RNA.

NGS has made significant contributions to our knowledge of the mutational landscape of MM. In particular, *KRAS* and *NRAS* mutations have been identified in approximately 20% of patients [37,38,39]. Importantly, the *DIS3* and *FAM46C* genes were first discovered as tumor suppressor genes relevant to MM by NGS, which identified these lesions in approximately 7% and 9% of cases, respectively [37,38,39]. NGS has also been utilized to identify two mutational signatures in MM: signature A involving cytosine-thymidine mutations at CpG islands and signature B associated with dysfunctional apolipoprotein B mRNA editing enzyme catalytic polypeptides such as APOBEC (apolipoprotein B mRNA editing catalytic polypeptide-like) [40]. The role of these mutations in the pathogenesis of MM and their prognostic significance are yet to be determined. It is also possible to detect the extent of genome-wide loss of heterozygosity (LOH), which might be related to homologous recombination deficiency (HRD), by using NGS. Moreover, this has been shown by Pawlyn et al. to be associated with impaired outcomes [41].

Clonal evolution is an important concept in cancer biology and was shown to be applicable in MM using NGS performed at serial time points in the patient’s disease course [42]. Improved understanding of clonal dynamics is likely to have an impact on the prognostication and treatment of MM in the future. The field where NGS is closest to routine clinical application is in the detection of minimal residual disease (MRD) [43]. NGS-based quantification of clonotypic immunoglobulin gene rearrangements is the basis for this technique, which will be discussed in detail in the subsequent section [44]. In summary, NGS in MM is evolving rapidly and has great potential to influence the diagnosis, risk stratification, treatment, and response monitoring of MM in the near future.

The molecular profiling of MM has seen major advances over the past decade. Although the techniques in routine clinical use at present are karyotyping and FISH, it is likely that the more sensitive techniques with higher resolution discussed above will play an increasingly important role. There is little doubt that further advances in techniques analyzing the MM genome will become available in the near future, and among these is an appreciation of three-dimensional genome organization [45]. Important considerations when applying these novel techniques to clinical practice include inter laboratory standardization and stringent quality control [46].

## 3. Newer Genomic Techniques

### 3.1. Liquid Biopsies

MM is a multifocal disease that is known for spatial genomic heterogeneity, with genetically different cancer sub-clones being located at different sites of disease in an affected individual. Therefore, a traditional bone marrow (BM) biopsy where only one site is sampled may not truly reflect the entire mutational profile in MM. Another consideration is the ability to perform serial monitoring as the genomic landscape of MM patients may evolve over time and treatment exposure and may inform treatment decisions. However, in clinical practice, bone marrow assessment is usually conducted at diagnosis and rarely repeated at remission or relapse. Repeated bone marrow biopsies are associated with pain and discomfort, not to mention increased risk of bleeding and infection. Therefore, a less invasive method of serial monitoring would be preferable.

“Liquid biopsy” is a simple and non-invasive alternative to surgical biopsies which enables the mutational profiling of a tumor at diagnosis, serial monitoring of response, and identification of biomarkers. This term applies to circulating tumor cells (CTCs), cell-free DNA (cfDNA), exosomes, circulating miRNAs, and long noncoding RNAs.

While originating in the BM, MM tumor cells are able to migrate into the peripheral blood stream, where they can be isolated and characterized. Various techniques have been used to identify CTCs, from the early days of immunofluorescence to allele-specific oligonucleotide polymerase chain reaction (ASO-PCR). However, flow cytometry made the identification of CTCs significantly easier and made it easier to adopt into clinical practice. Studies have shown that CTCs are distinct from BM myeloma tumor cells and have unique phenotypic and cytogenetic features lending credence to spatial heterogeneity [47].

Clinically, CTCs are able to detect active disease even at low levels [48,49]. Studies have shown that CTCs have prognostic value in relapsed disease, with the presence of high numbers of CTCs predicting worse survival in patients with active disease relapse, as CTCs correlate with disease that has higher proliferation, adverse cytogenetics, and shorter time to next treatment [50,51,52].

Detectable CTCs at the time of autologous stem cell transplant (ASCT) have been used to stratify MM patients undergoing ASCT and is an independent prognostic factor for time to progression (TTP) and overall survival (OS) [53,54]. Even in this era of novel agents and ASCT, the investigators of the GMMG-MM5 trial demonstrated that the reduction of detectable CTCs was positively correlated with clinical response [55]. However, this study also demonstrated that there is discordance between the BM and peripheral blood (PB) samples, indicating that CTCs may not be adequate to assess MRD [53].

Even in smoldering multiple myeloma (SMM), CTCs are able to risk stratify patients and identify patients at high risk of progression. High levels of CTCs (defined as absolute peripheral blood plasma cells > 5 ×10^6^/L and/or >5% plasma cells per 100 cytoplasmic immunoglobulin (Ig)-positive peripheral blood mononuclear cells) are able to identify patients with SMM that are at high risk of progression to MM (i.e., 71% and 86% progressing to MM within 2 and 3 years, respectively) [56]. This study was replicated using flow cytometry with the same observation that the presence of CTCs in SMM patients predicts a shorter median TTP (10 months vs. not reached) and a higher 2-year probability of progression (58% vs. 9%) to MM [57].

Another liquid biopsy assessment method is cell-free DNA. CfDNA is isolated from the blood plasma of cancer patients, which contains tumor-derived DNA fragments that are shed into the blood stream by cancer cells. Assessment of cfDNA using next-generation sequencing correlates with active disease, with higher levels present in MM patients compared to normal volunteers and non-MM cancers [58]. The cfDNA levels also correlate with different clinical stages of plasma cells tumors ranging from monoclonal gammopathy of undetermined significance (MGUS), to smoldering MM and symptomatic MM. The amount of cfDNA correlates with the disease stage and international staging system (ISS) stage but may not be able to differentiate between newly diagnosed and relapsed MM [59].

It has now been shown that there is good correlation between blood and bone marrow samples. The sensitivity rates of cfDNA in harbouring clonal and subclonal mutations present in the bone marrow were 100% and 96%, respectively [60]. Although there is good correlation, there are studies which identified mutations such as *PIK3CA* that are found only in the plasma and not in the BM. Similar to CTCs, this phenomenon supports the existence of spatial and clonal heterogeneity in MM [58,61].

MM patients with detectable cfDNA had poor response to treatment and relapsed earlier compared to patients with low/undetectable cfDNA [44,58,61,62]. However, all of these studies were limited by small sample sizes and a lack of homogenous treatment and monitoring strategies.

There still exist limitations to using cfDNA, with conflicting studies regarding the efficacy of using cfDNA to identify mutations. Mithraprabhu et al. demonstrated in their study that when profiling paired PB and BM samples, 46% of the mutations were found only in BM and 24% were found only in PB [58]. The authors speculated as to whether using enriched CD138+ MM cells caused a higher detection rate as opposed to cfDNA, which had a mixture of normal and tumor cells. However, this may indicate spatial heterogeneity, where a discordance between the marrow and blood is expected. Further studies are needed to elucidate this phenomenon.

Another limitation of the two techniques is that both entities are scarce in circulating biofluids and may be inadequate as clinically applicable diagnostic biomarkers. Consequently, other molecules derived from tumor cells, such as noncoding RNAs (ncRNAs) and exosomes, which are far more abundant than cfDNA or CTCs in biofluids and are relatively stable in a variety of biological fluids as well as frequently dysregulated even in the earliest stages of cancer, are being investigated for clinical use.

Exosomes are 50- to 140-nm membrane-bound particles that originate from large multivesicular bodies (MVBs) and are released into the extracellular environment by the fusion of MVBs with the plasma membrane. Exosomes can also be released in large quantities in various biological fluids, such as plasma, urine, saliva, ascites, and bronchoalveolar lavage fluid. They contain proteins and nucleic acids, such as microRNAs (miRNAs) and mRNAs, and participate in genetic exchange between cells [63,64]. Tumor-derived extracellular vesicles (EVs) target various cell types to modify the tumor microenvironment, which supports the growth of tumor cells by inducing angiogenesis, tumor cell migration and metastasis, immune response modulation, and drug resistance. EVs derived from hematological tumor cells, such as MM cells, contribute to angiogenesis. Several pathways, such as the signal transducer and activator of transcription 3 (STAT3), c-Jun *N*-terminal kinase, and p53, are modulated by the EVs from MM in endothelial and bone marrow stromal cells (BMSCs).

Circulating miRNAs have emerged as appealing biomarkers because they can be evaluated from PB and have been reported as prognostic tools in many cancer types [65], including MM [64,66,67,68]. However, many circulating miRNAs are passively released from apoptotic and necrotic cells and therefore may not truly reflect the tumor cells’ biology. In contrast, exosomes are actively secreted in the PB by different cell types, including cancer cells, and are biologically relevant because they promote tumorigenesis through miRNA transfer [69].

MiRNAs also play a role in drug resistance. MiRNAs such as let-7b, let-7e, miR-106a, miR-106b, miR-155, miR-16, miR-17, miR-18a, and miR-20a were identified as significant predictors for progression-free survival (PFS) by inducing tumor cell resistance to bortezomib [70]. Downregulation of several miRNAs, including miR-16-5p, miR-15a-5p, miR-20a-5p, and miR-17-5p, was observed in the patients with bortezomib resistance [71]. Another study identified six miRNAs (miR-26a-5p, miR-29c-3p, miR-30b-5p, miR-30c-5p, miR-193a-5p, and miR-331-3p) that were significantly downregulated in poor responders to lenalidomide with low-dose dexamethasone treatment [36]. Even with ASCT treatment, let-7b and miR-18a were associated with poor outcomes with regard to PFS and OS [72].

Noncoding RNAs (ncRNAs) have been arbitrarily categorized into short and long ncRNAs (lncRNAs). Short ncRNAs can be further subdivided into various categories, including microRNAs (miRNAs), small interfering RNAs, PIWI-associated RNAs, and small nucleolar RNAs. Dysregulated short ncRNAs, especially miRNAs, are known to have important functions in virtually all types of cancer, including MM. Notably, miRNAs have also gained importance as innovative therapeutic targets in cancer, including MM [73,74,75]. Long noncoding RNAs have been of interest because, even at low level of expression, circulating cell-free lncRNAs are promising putative non-invasive diagnostic and prognostic biomarkers in cancer patients [76]. However, this is a relatively new area of research and caution is required when interpreting these findings.

In conclusion, liquid biopsy has been shown to be able to detect disease, monitor response to treatment, and stratify disease, and it also may be useful in MRD. The additional ability of these techniques to identify novel mutations and afford clinicians the ability to sequentially track response to therapy may change the way we diagnose and monitor MM patients in the future.

### 3.2. Single-Cell Genomics

One major advantage of profiling single cells (whether at the genomic, protein, or metabolite level) over bulk samples is the elucidation of clonal heterogeneity. Clinically meaningful information in this context concerns the identification of subclonal populations that (1) might predict disease outcome (e.g., genomically complex clones that may correlate with more aggressive disease) and (2) correlate with drug sensitivity or resistance [77].

In MM (and more broadly in the hemato-lymphoid neoplasm arena), the examples of established clinical single-cell analysis methods include cytogenetics (karyotyping and FISH) and flow cytometry. Karyotyping, while providing clinically valuable information (for example, the identification of ploidy status and clonal evolution), suffers from the drawback of being a low-throughput assay; only 20 cells are analyzed routinely. While more cells (up to 100) may be analyzed with FISH, this technique only allows for the detection of a priori defined targets [6].

Flow cytometry is a higher-throughput technology that enables profiling of tens of thousands of cells. The major limitation of flow cytometry is the number of markers (or parameters) that can be profiled simultaneously for an individual cell —currently, this number does not exceed 20 [78]. Furthermore, unless cancer mutation-specific detection methods are developed [79], flow cytometry does not enable the definitive identification of neoplastic cells.

Single-cell genomics, more specifically defined as genome or transcriptome sequencing at the single-cell level, has in theory the following advantages over the aforementioned methods: (1) significantly higher throughput compared to cytogenetic analysis (modern platforms have throughputs comparable to flow cytometry) [80]; (2) vastly greater number of parameters (in the thousands) can be analyzed per cell compared to flow cytometry; and (3) the identification of mutations at base-pair resolution, which by extension would mean the definitive identification of neoplastic cells.

There have been recent studies reporting the results of single-cell genomic analysis in MM. Lohr et al. [81] performed RNAseq (transcriptomic) and DNAseq (targeted) on single circulating MM cells. DNAseq enabled the detection of *KRAS* and *NRAS* mutations in MM circulating tumor cells (CTCs) that had been previously identified on clinical-grade bulk bone marrow sequencing. Expression analysis from single-cell RNAseq data was able to discriminate between normal plasma cells and MM cells, and could also be used to infer the existence of MM chromosomal translocations in CTCs. This study provided proof-of-concept concerning the feasibility of MM CTC genomic analysis.

Mitra et al. [82] performed single-cell targeted gene expression profiling on human myeloma cell lines and single-cell RNAseq data of patient primary bone marrow MM cells. Machine learning algorithms were then used to predict resistant cells within the primary MM population using the single-cell targeted gene expression data.

Several challenges exist in the routine clinical implementation of single-cell genomic profiling [83]. The known technical challenges are significant stochastic loss of polyadenylated RNA during sample preparation and amplification bias, especially for lowly expressed genes [84]. From a clinical laboratory perspective, standardization and simplification of the workflow, both for the wet-bench and bioinformatics analytical components, will be critical to ensure accuracy and reproducibility.

In summary, single-cell genomic profiling is a highly promising technology that has immense potential in MM diagnostics. Although speculative at this juncture, we opine that its major advantages over existing diagnostic modalities include a more comprehensive analysis of an individual patient MM’s genomics landscape and the prediction of chemosensitivity profiles in single MM cells with the possibility of identifying drug-specific resistant subclones. Each technique has its own advantages and disadvantages that we have summarized in Table 1.

## 4. Clinical Utility of Genomics in Myeloma

### 4.1. Classification

For many years, studies have shown that MM is not a single disease entity. It is heterogeneous with different characteristics and genetic aberration plays an important role in this heterogeneity. Microarray technologies have allowed large numbers of genes to be evaluated and have helped to advance the development of molecular classification strategies in MM [85].

One such classification system is the translocation/cyclin D (TC) classification proposed by Bergsagel et al., which categorized MM by GEP into eight TC groups (11q13, 6p21, 4p16, maf, D1, D1+2, D2, and none) based on recurrent translocations, specific trisomies, and cyclin D genes expression. Interestingly, these groups were shown to have significant differences in terms of prevalence of bone disease, frequency of relapse, and progression to extramedullary tumor [86].

The University of Arkansas for Medical Sciences (UAMS) group classified MM into seven subgroups based on common gene expression signatures: PR (proliferative), LB (low bone disease), MS (*MMSET*), HY (hyperdiploid), CD-1 and CD-2 (based on *CCND1* and *CCND3* expression), and MF (*MAF/MAFB*). These subgroups were strongly influenced by known genetic lesions, such as *c-MAF*- and *MAFB*-, *CCND1*- and *CCND3*-, and *MMSET*-activating translocations and hyperdiploidy [87].

In addition to the seven subgroups defined by the UAMS group, Broyl et al. from the HOVON-65/GMMG-HD4 trial group identified another three novel subgroups: the group with high expression of nuclear factor kappa light chain enhancer of activated B cells pathway genes (NF-κB cluster), the group with overexpression of cancer testis antigens without overexpression of proliferation genes (CTA cluster), and the group with upregulated protein tyrosine phosphatases PRL-3, PTPRZ1, and SOCS3 (PRL3 cluster) [88].

Chng et al. also specifically evaluated the hyperdiploid subtype of MM by GEP and showed that this particular MM subtype exhibited the overexpression of genes involved in protein biosynthesis. These findings allowed the patients to be divided into four clusters based on the expression of largely mutually exclusive genes, in which cluster 1 was associated with cancer testis antigen genes and mitosis/proliferation-related genes; cluster 2 was associated with hepatocyte growth factor (*HGF*) and interleukin-6 (*IL-6*) genes; cluster 3 was associated with high expression of NF-κB and antiapoptotic genes; and cluster 4 was less defined but lacked high expression of the abovementioned genes and exhibited especially low expression of *HGF*. Clinical correlations were also observed with these clusters. Cluster 1 was noted to be more proliferative and cluster 3 was noted to have better response to bortezomib [89].

The development of these classification systems is important, especially in identifying high-risk genetic groups and for therapeutic implications [85].

### 4.2. Prognosis

Prognostication in MM has evolved over time, from the Durie-Salmon staging system, the ISS, and the most recent revised ISS (R-ISS), which is now being widely used. There have been significant developments in GEP, which has helped to improve the understanding of the disease biology of MM, also leading to the development of several prognostic and predictive gene signatures. While significant, the best way to incorporate these gene signatures into the treatment algorithm for MM remains an area of ongoing research.

The UAMS group used GEP to identify a 70-gene signature that was associated with short survival: 51 had high expression and 19 had low expression. The calculated risk score, which was derived from the log_2_-scale up- versus downregulated mean expression ratio, revealed 13% high-risk patients who had poorer event-free survival (EFS) and OS. Interestingly, 30% of the genes were mapped to chromosome 1 with most upregulated genes located in chromosome 1q, and downregulated genes located in chromosome 1p. This gene signature was reduced to a 17-gene signature with a similar OS estimate [90].

A study from the Intergroupe Francophone du Myelome(IFM) from France showed that the overexpression of genes involved in mitosis was associated with high-risk disease while hyperdiploid signatures were associated with low-risk disease. The IFM 15-gene model was proposed to identify high-risk patients with a 6.8-fold hazard ratio of death as compared to low-risk patients. Combined with SB2M ≥ 5.5 mg/L and/or t(4;14), it could even more accurately identify the high-risk group of patients [91].

A study from Europe using human myeloma cell lines (HMCL) identified seven genes which had poor prognostic value for both EFS and OS. This HMCL score categorized patients into low, intermediate, and high risk [92].

Chromosome instability, especially in the form of aneuploidy, is a hallmark in MM. Extra copies of the centrosome results in the formation of multipolar spindles, leading to chromosome mis-segregation and aneuploidy. While centrosomes are absent in most normal plasma cells, they are present in the majority of clonal plasma cells in all stages, including MGUS, and the percentage increases with the progression of stage, e.g. from MGUS to SMM. As high levels of centrosome amplification was shown to be associated with short survival, a gene expression-based Centromere Index (CI), which was calculated by adding the normalized expression value of the expression levels of genes encoding for the proteins in the centrosomes, was proposed by Chng et al. and it was noted that CI increased significantly from MGUS to MM. A CI of more than 4 was highly correlated with centrosome amplification by immunofluorescence and was associated with short survival [93,94].

Chromosome instability genome event count (CINGEC), a novel measure of chromosomal instability, was introduced. It is an algorithm that incorporates both copy number aberrations and interstitial breakpoints and was shown to be an independent prognostic factor in MM associated with disease progression and survival. CINGEC-associated gene expression profile signature, CINGECS, which consisted of genes involved in DNA damage responses in addition to aneuploidy and other generic oncogenic processes, had survival association [95].

Homozygous deletions (HZD) has been found to inactivate genes and it has been shown that there was significant enrichment of cell death network genes in the list of genes with HZD identified. Dickens et al. identified 97 genes annotated as cell death, and showed that there were two distinct clusters with different expression patterns, with the higher-expressing cell death cluster having a poorer prognosis. For transition to clinical use, a six-gene cell death signature was derived, using the ratios of expression of three pairs of genes. A ratio of ≥1 in any pair was associated with poor prognosis [96].

A prognostic signature consisted of 92 genes (EMC-92-gene signature) was shown to be able to identify high-risk patients with reduced OS. In further study, known published risk markers in MM were evaluated and the combinations of FISH, ISS, and GEP were evaluated to determine the strongest predictor for OS. ISS-GEP combinations consistently showed the strongest prediction, with EMC92-ISS compound risk marker, combining EMC92 and ISS, having the best median rank score. EMC92-ISS is particularly useful because it can be used to identify both high- and low-risk MM patients [97,98].

With the multitude of GEP options, IMWG performed detailed computational simulations to investigate the integration of proven prognostic signatures for improved patient risk stratification. The gene signatures included were as described above: chromosome instability signature (CINGECS), centrosome index (CNTI), prognostic signature from MM cell line study (HMCL), cell death signature implicated by homozygous deletion (HZDCD), 15-gene prognostic signature by the Intergroupe Francophone du Myelome 99 clinical trial (IFM15), proliferation signature (PI), and 70- and 80-gene prognostic signatures by UAMS researchers (UAMS70, UAMS80). The study identified the EMC92 + HZDCD combination as the top-performing prognostic signature combination across all datasets, which was associated with the best improved performance in terms of hazard ratio and *p*-value [99].

Despite many emerging gene signatures being developed, the best way to incorporate these GEP results into clinical practice remains unclear. There have been ongoing studies looking at combining GEP with known prognostic models. One of the first scoring systems incorporating GEP was ISS-MUT, combining copy number and structural abnormalities (CNSAs) and the poor prognostic mutation (MUT) with ISS. The adverse CNSAs were t(4;14), del(17p), *MYC* translocations, and amp(1q). The poor prognosis mutations included: *TP53*, *ZFHX4*, *CCND1*, *ATM*, and *ATR*. ISS-MUT divided the patients into three groups: group1, ISS I and II with no lesion; group 2, ISS III with no lesion or ISS I, II, and III with one lesion; and group 3, two lesions regardless of ISS. ISS-MUT was able to identify the cohort with early progression and early mortality, in addition to groups with different OS and PFS [39].

### 4.3. Predictive Markers And Treatment Selection

The findings from GEP have led to a better understanding of the genomics and disease biology in MM. Based on this knowledge, there has been increased research looking into the development of mutation-directed therapy to improve patient outcome. Some pathways and targeted therapies identified include the inhibition of mitogen-activated protein kinase (MAPK) kinase (MEK) with trametinib [100,101], the use of vemurafenib in MM patients with the *BRAF* mutation [102], and targeting the apoptotic pathway with venetoclax, especially in MM patients with t(11;14) mutation, where there is a high level of BCL-2 expression [103].

In addition, as described earlier, high CI is an independent prognostic factor associated with short survival. Further study by the same group showed that tumors with high CI particularly overexpressed *STK6* (coding for protein aurora-A) and *AURKB* (coding for protein aurora-B), which mediate centrosome amplification. HMCL with higher CI were more responsive to treatment with a novel aurora kinase inhibitor and this suggests that aurora kinase might represent a novel therapeutic target in this cohort with poor prognosis [94].

### 4.4. Minimal Residual Disease (MRD)

With advances in the treatment of MM, there has been increasing focus on achieving deeper responses following MM treatment, and MRD monitoring has been seen as an important target in the treatment algorithm for MM. Munshi et al. showed in their meta-analysis that the achievement of MRD negativity was associated with a significant improvement in PFS and OS [104]. In addition, a pooled analysis of three PETHEMA/GEM clinical trials showed similar findings as well [105].

There have been various techniques developed to detect MRD by using bone marrow samples, including multi-parametric flow cytometry (MFC) with a sensitivity level of 10^4^ to 10^5^ [106] and allele-specific oligonucleotide quantitative PCR (ASO-qPCR), which has been shown to be a powerful technique to assess MRD [107]. There have also been developments to improve the sensitivity of MRD detection including next-generation flow cytometry and next-generation sequencing to detect clonal immunoglobulin VDJ gene rearrangements for even better sensitivity in detecting myeloma cells [108]. By using the standardized EuroFlow technique, next-generation flow cytometry is able to reach a sensitivity level of 10^5^ or even 10^6^. The NGS technology is able to reach a sensitivity level of 10^6^ and is well standardized [109]. Comparison between the two techniques showed good concordance with only a few discrepant cases [109].

The level of sensitivity we should aim to achieve remains an ongoing discussion. In the latest IMWG consensus criteria, MRD was included as part of the MM response criteria and requires a minimum sensitivity of 1 in 10^5^ nucleated cells or higher [110]. The IFM/DFCI 2009 study showed that 10^6^ should be the optimal cutoff, as patients with and without that level of detectable disease shown very significant differences in PFS [111]. Currently, most studies with adequate follow-up show that negative MRD does not equate to a cure in myeloma. This could well be due to the fact that the level of sensitivity used in these studies is 1 in 10^5^ or poorer. To identify patients that could potentially be cured, a deeper level of MRD at 1 in 10^6^ cells may be necessary.

In addition, there has also been growing interest in the detection of MRD from peripheral blood given the inherent limitations of bone marrow biopsies for disease assessment in MM. Bone marrow biopsies evaluate a single focal point, and hence may not be able to comprehensively assess the multifocal nature of MM. In addition, it is also impractical to perform multiple repeated bone marrow biopsies in clinical practice. The detection of circulating tumor DNA (ctDNA) and exosomal microRNAs (miRNAs) as well as more sensitive methods of M-protein detection are some of the advances in this field. Spencer’s group from Australia showed that ctDNA analysis is an important adjunct to bone marrow biopsy to improve response monitoring in MM [58]. Ghobrial’s group from the Dana-Farber Cancer Institute showed that circulating exosomal miRNAs enhances the stratification of patients with high-risk factors [112]. It might potentially be the marker to be used in the future for MRD assessment. The Mayo Clinic group evaluated a more sensitive way of detecting M-protein as compared to the standard immunoelectrophoresis, miRAMM (monoclonal Ig rapid accurate mass measurements), which is based on the accurate molecular mass of light chain components. This enables the detection of MRD in deep response, which might otherwise be unable to be detected by the traditional immunoelectrophoresis [113].

Moving forwards, MRD will likely play an important role in the myeloma treatment algorithm. More work is necessary, however, to determine the optimal MRD method and appropriate time points for testing before MRD can be incorporated into risk-adapted therapy.

## 5. Conclusions and Future Direction

There are multiple methods for the detection of genomic abnormalities in myeloma. These methods have pros and cons and need to be selected based on their utility. Most commonly used is the FISH method for risk stratification. However, it is possible that NGS-based platforms could replace FISH in detecting translocation and copy number abnormalities as well as allow for the identification of gene mutations. Currently, the main utility of genomics is in risk stratification. However, increasingly, with the development of therapies such as venetoclax and *BRAF* inhibition which benefit patients with specific genetic abnormalities in MM, the future utility of genomics could include treatment selection as well as the assessment of MRD. In terms of MRD, liquid-based detection may allow for a better representation of the spatial heterogeneity in MM, overcome the practical limitations of bone marrow-based MRD assessment, and allow for more frequent assessments to be conducted. The current limitation is technical and related to sensitivity but one may foresee that in the future, MRD assessment could well be blood-based.

## Figures and Tables

**Table 1:** Advantages and disadvantages of different genomic techniques.

Techniques	Advantages	Disadvantages
Metaphase Karyotyping	- Able to identify numerical as well as structural chromosomal abnormalities - Low cost - Widely available	- Requires the presence of plasma cells in metaphase - Karyotypic abnormalities are only present in 25–30% of multiple myeloma (MM) patients
Fluorescent in Situ Hybridization (FISH)	- Able to identify known recurrent abnormalities - High sensitivity - Applicable in interphase plasma cells	- Only allows for the detection of a priori defined targets and unable to identify novel or unexpected genetic changes
Gene Expression Profiling (GEP)	- Able to assess the transcriptome of cancer cells, allowing the identification of changes in gene expression which are independent of changes in sequence, e.g. epigenetic silencing.	- Not as good as RNA sequencing (RNASeq) in detecting splicing variants, noncoding RNA, and fusion proteins - Significant cost - Expertise required for interpretation - Standardization of methodology in progress
RNA Sequencing (RNASeq)	- Same as those for GEP - Able to detect splicing variants, noncoding RNA, and fusion proteins better than GEP	- Significant cost - Expertise required for interpretation - Standardization of methodology still in progress
Array Comparative Genomic Hybridization (ACGH)	- Able to assess genome-wide copy number changes in cancer - Great sensitivity for detecting genomic imbalances	- Not as good as FISH in detecting IgH translocations - Significant cost - Expertise required for interpretation - Standardization of methodology still in progress
Next-Generation Sequencing (NGS)	- Able to detect abnormalities across the entire genome - Greater sensitivity compared to karyotyping and FISH - Reduced turnaround time as a result of massively parallel sequencing and a smaller volume of the test specimen required as compared to Sanger sequencing	- Significant cost - Expertise required for interpretation - Standardization of methodology still in progress
Circulating Tumor Cells (CTC)	- Able to evaluate mutational profiling of tumor by using peripheral blood sample - May be able to overcome issue with spatial heterogeneity in MM	- Sample maybe inadequate
Cell-free DNA (cfDNA)	- High degree of cancer specificity - May be able to overcome issue with spatial heterogeneity in MM	- Sample may be inadequate - Conflicting studies regarding the efficacy of using ctDNA to identify mutation
Exosomes	- Good sensitivity	- Long preparation and technically difficult processing
Circulating miRNA	- Offers superior sensitivity and specificity compared with ctDNA for diagnosing colorectal cancers	- Lack of disease and organ specificity and uncertainty of normalization
Circulating cell-free long noncoding RNA	- Good sensitivity	- High cost - Paucity of data to validate findings
Single-Cell Genomics	- Significantly higher throughput - Greater number of parameters (in the thousands) can be analyzed per cell - Identification of mutations at base-pair resolution, which by extension would mean the definitive identification of neoplastic cells	- Risk of significant stochastic loss of polyadenylated RNA during sample preparation and amplification bias, especially for lowly expressed genes
